# Predictors of long-term cancer-related distress among female *BRCA1* and *BRCA2* mutation carriers without a cancer diagnosis: an international analysis

**DOI:** 10.1038/s41416-020-0861-3

**Published:** 2020-05-12

**Authors:** Kelly A. Metcalfe, Melanie A. Price, Carol Mansfield, David C. Hallett, Geoffrey J. Lindeman, Angie Fairchild, Joshua Posner, Sue Friedman, Carrie Snyder, Kathleen Cunningham Foundation Consortium for Research into Familial Breast Cancer, Henry T. Lynch, D. Gareth Evans, Steven A. Narod, Alexander Liede

**Affiliations:** 1 Women’s College Research Institute, Women’s College Hospital, Toronto, ON Canada; 2 Lawrence S. Bloomberg Faculty of Nursing, University of Toronto, Toronto, ON Canada; 3 Centre for Medical Psychology and Evidence-based Decision-making (CeMPED), School of Psychology, The University of Sydney, Sydney, NSW Australia; 4 RTI Health Solutions, Research Triangle Park, NC USA; 5 Center for Observational Research, Amgen Inc., Thousand Oaks, CA USA; 6 The Royal Melbourne Hospital and Peter MacCallum Cancer Centre, Melbourne, VIC Australia; 7 The Walter & Eliza Hall Institute of Medical Research, Melbourne, VIC Australia; 8 Facing Our Risk of Cancer Empowered (FORCE) Advocacy Organization, Tampa, FL USA; 9 Creighton University, Omaha, NE USA; 10 Kathleen Cuningham Foundation Consortium for Research into Familial Breast Cancer (kConFab), Research Department, The Sir Peter MacCallum Cancer Center, University of Melbourne, Parkville, VIC Australia; 11 Manchester Centre for Genomic Medicine, University of Manchester, Manchester, UK; 12 Center for Observational Research, Amgen Inc., South San Francisco, CA USA

**Keywords:** Cancer prevention, Predictive markers, Breast cancer

## Abstract

**Background:**

Women with a *BRCA1* or *BRCA2* mutation have high lifetime risks of developing breast and ovarian cancers. We sought to estimate the prevalence of cancer-related distress and to identify predictors of distress in an international sample of unaffected women with a *BRCA* mutation.

**Methods:**

Women with a *BRCA1/2* mutation and no previous cancer diagnosis were recruited from the United States, Canada, the United Kingdom, Australia and from a national advocacy group. Using an online survey, we asked about cancer risk reduction options and screening, and we measured cancer-related distress using the Impact of Event Scale.

**Results:**

Among 576 respondents, mean age was 40.8 years (SD = 8.1). On average 4.9 years after a positive test result, 16.3% of women reported moderate-to-severe cancer-related distress. Women who had undergone risk-reducing breast and ovarian surgery were less likely to have (moderate or severe) cancer-related distress compared to other women (22.0% versus 11.4%, *P* value = 0.007). Women recruited from the advocacy group were more likely to have cancer-related distress than other women (21.6% versus 5.3%, *P* value = 0.002).

**Conclusions:**

Approximately 16% of women with a *BRCA1* or *BRCA2* mutation experience distress levels comparable to those of women after a cancer diagnosis. Distress was lower for women who had risk-reducing surgery.

## Background

Since testing for *BRCA1* and *BRCA2* began in the 1990s, there has been keen interest in the psychosocial consequences of genetic testing.^1–6^ The risk of developing breast cancer by age 80 years is ~72% for *BRCA1* mutation carriers and 69% for *BRCA2* mutation carriers; the risk for ovarian cancer is 44% for *BRCA1* carriers and 17% for *BRCA2* carriers.^7^ Healthy women with a mutation are given information about these risks and have the options of risk-reducing surgery and screening. It is anticipated that receipt of this genetic information will be stressful for many women, but it is not clear how many experience significant distress, nor the duration of distress.^4,6^ The majority of psychosocial research in women with a *BRCA1* or *BRCA2* mutation suggests that distress increases immediately following the receipt of positive genetic test results, but return to baseline levels or below with time.^4,6^ However, the majority of research has focussed on women from academic genetic testing clinics, has included women with negative and positive BRCA results and has included women with and without a cancer diagnosis.

It is important to assess psychosocial functioning in the period following genetic testing for women without a cancer diagnosis. At this time, women face difficult decisions about cancer risk reduction. Several surveillance and prevention options are available with the goals of early detection and of reducing cancer incidence and mortality. Furthermore, the practice of genetic testing for *BRCA1* and *BRCA2* has changed over the past 20 years. Genetic testing can now be obtained directly by the consumer or ordered by the treating physician without genetic consultation. Further, there is increasing interest in population-based genetic testing for *BRCA1* and *BRCA2* with the goal of identifying women with a mutation prior to cancer diagnosis.^8^ In these situations, the genetic counsellor is often bypassed. It is important to assess the benefits and the risks of new genetic testing protocols, in particular among healthy women who receive a positive test result. These risks many include acute and chronic psychological distress. In the current study, we report on frequency and predictors of cancer-related distress in a large international cohort of unaffected women with *BRCA1* or *BRCA2* mutations aged 25 to 55 years.

## Methods

### Participants and procedures

Eligible women were unaffected by cancer, aged 25 to 55 years, and were *BRCA1* or *BRCA2* mutation carriers. All were able to read and understand English and consented to participate in an international patient preferences study around risk-reducing strategies for familial/genetic risk of developing breast and ovarian cancer.

Recruitment was between January 2015 and March 2016 via six sources across the United States, Canada, United Kingdom and Australia, including the patient advocacy group Facing Our Risk of Cancer Empowered (FORCE), clinical research registries at Creighton University (USA), Women’s College Hospital (Canada), The Royal Melbourne Hospital (Australia), the Kathleen Cunningham Foundation Consortium for Research into Familial breast cancer (kConFab) at Peter MacCallum Cancer Centre (Australia) and Manchester Centre for Genomic Medicine (UK). FORCE respondents provided a self-reported *BRCA1/2* status and were recruited through its website, newsletters and social media. Clinical sites identified respondents who met the inclusion criteria and mailed them invitation letters with the URL of the online survey and a unique password. Institutional review boards at RTI International and all participating sites approved the study. All participants provided informed consent prior to their inclusion in the study.

Participation involved completing an anonymous online survey developed following good research practices.^9^ The questions were developed with input from clinicians who treat women with *BRCA1/2* mutations. The survey instrument was pretested in 14 one-on-one interviews with women in the United States who met the study inclusion criteria to assess respondent comprehension, the relevance of the questions to respondents and survey flow.^10^ Questionnaires assessed demographic, clinical (uptake of cancer screening, risk-reducing surgery and chemo-prevention) and genetic data (*BRCA1/2* mutation status, date of testing). In addition, participants were asked to complete a family history questionnaire and the Impact of Event Scale (IES).^11^ The family history questionnaire asked about first- and second-degree relatives (with definitions) diagnosed with breast cancer before age 50 years, ovarian cancer at any age, male breast cancer, bilateral breast cancer and three or more breast cancer at any age, a combination of breast, ovarian and/or pancreatic cancer on the same side of the family.

The IES was used to measure cancer-related distress. The event was “Being at increased risk of cancer because of a confirmed mutation in the *BRCA1* or *BRCA2* genes”. For each item, respondents were asked to indicate how frequently each item was true for them during the past 7 days, with the answer choices being “Not at all”, “A Little Bit”, “Moderately” and “Quite a Bit”. Total distress scores can range between 0 and 75. Scores between 0 and 8 are considered sub-clinical, between 9 and 25 indicate mild distress, between 26 and 43 indicate moderate distress and scores >43 indicate severe distress.

### Statistical methods

Descriptive statistics were used to characterise the sample. Multivariable logistic regression models were used to assess differences between women with sub-clinical or mild distress and women with moderate-to-severe psychological distress. Regression modelling was conducted for all respondents. Covariates included were based on a priori understanding of cancer-related distress work: age (40 years and older or younger than 40 years), whether a first-degree relative has ever been diagnosed with breast cancer and ovarian cancer (yes or no), higher education level (4-year college or higher or no 4-year college degree), marital status (yes or no), years since gene identification (continuous years), whether the respondent has children (yes or no), whether the respondent has had risk-reducing surgery (risk-reducing bilateral mastectomy (RRBM), bilateral salpingo oophorectomy (BSO), both or none) and recruiting source (online through FORCE or through a clinic). Tests of association between respondents who were recruited through clinics and respondents who were recruited through FORCE were calculated using two-sample *t* tests for sample means, *χ*^2^ tests (frequencies >5 in each category) and Fisher’s exact test (frequencies <5 in at least one category) for categorical variables. The multivariable logistic regression models were generated using the SAS software, version 9.4 (SAS Institute Inc., Cary, NC). Summary statistics and associated *P* values were generated using the Stata, version 15 software (StataCorp, College Station, TX). All *P* values < 0.05 (two-tailed) were considered to be statistically significant.

### Patient sample

Between January 2015 and March 2016, subjects were recruited through international clinical sites and online through the advocacy group (FORCE). The clinical sites mailed 1163 letters to potentially eligible women, 383 women accessed the survey, and 338 met the inclusion criteria. Of the women who met the inclusion criteria, 303 completed the IES questions in the survey and provided data on the covariates used for analysis. Through FORCE (advocacy group), 1374 women accessed the survey, and 494 met the inclusion criteria. Of the women who met the inclusion criteria, 273 completed the IES questions in the survey and provided data on the covariates used for analysis. Combining the women recruited through clinics (*n* = 303) and recruited online through FORCE (*n* = 273), the final sample size was 576.

## Results

Of the 576 study participants, 52.3% had a BRCA1 mutation, 45.1% had a BRCA2 mutation, and 1.4% had both a BRCA1 and BRCA2 mutation (1.2% were unsure of which gene). The mean age at the time of questionnaire completion was 40.8 years (SD = 8.1) and the mean time elapsed since genetic testing was 4.9 years (SD = 4.4, range 0–23 years). The majority of participants were from the United States (54.0%), but others were from the United Kingdom (20.3%), Australia (20.3%) and Canada (5.4%) (Table 1). Of the 311 USA participants, 273 were recruited from FORCE and 38 were recruited from Creighton University. Table 1 presents the characteristics of women recruited through a clinic and through FORCE separately. Looking at some of the larger differences, the FORCE sample had a higher percentage of college educated women and women employed full time. The average time since diagnosis was shorter in the FORCE sample.

**Table 1:** Summary statistics.

Source of recruitment	Summary statistics			
	Number of respondents (%)^a^, except where noted			
	Clinic (*n* = 303)	FORCE (*n* = 273)	Test of difference*, P* value	All respondents (*N* = 576)
Age (years)				
Min, max	25, 55	25, 55	0.599	25, 55
Mean (SD)	40.68 (8.11)	41.04 (8.16)		40.85 (8.13)
Median	40	41		41
Age category				
25‒39	137 (45.2%)	117 (42.9%)	0.569	254 (44.1%)
40‒55	166 (54.8%)	156 (57.1%)		322 (55.9%)
Ethnicity				
White or Caucasian	282 (93.1%)	257 (94.1%)	0.136	539 (93.6%)
Black or African decent	0 (0.0%)	3 (1.1%)		3 (0.5%)
Hispanic or Latino	1 (0.3%)	3 (1.1%)		4 (0.7%)
Asian	5 (1.7%)	3 (1.1%)		8 (1.4%)
Other	15 (5.0%)	7 (2.6%)		22 (3.8%)
Higher education (4-year college and higher)	170 (56.1%)	217 (79.5%)	<0.001	387 (67.2%)
Marital status				
Married/living as married/civil partnership	233 (76.9%)	207 (75.8%)	0.834	440 (76.4%)
Single/never married	42 (13.9%)	43 (15.8%)		85 (14.8%)
Divorced/separated/widowed/other	28 (9.2%)	23 (8.4%)		51 (8.9%)
Have child or children	59 (19.5%)	56 (20.5%)	0.755	398 (69.1%)
Employment status				
Employed full time	159 (52.5%)	170 (62.3%)	0.003	329 (57.1%)
Employed part time	74 (24.4%)	32 (11.7%)		106 (18.4%)
Self-employed	28 (9.2%)	28 (10.3%)		56 (9.7%)
Homemaker	23 (7.6%)	32 (11.7%)		55 (9.5%)
Other	19 (6.3%)	11 (4.0%)		30 (5.2%)
Income (above median income in respective country)	136 (44.9%)	75 (27.5%)	<0.001	211 (36.6%)
Country				
US	38 (12.5%)	273 (100.0%)	─	311 (54.0%)
UK	117 (38.6%)	─		117 (20.3%)
Australia	117 (38.6%)	─		117 (20.3%)
Canada	31 (10.2%)	─		31 (5.4%)
Place of recruitment				
Clinic	303 (100.0%)	─	─	303 (52.6%)
Online through FORCE	─	273 (100.0%)		273 (47.4%)
Mutation				
* BRCA1*	154 (50.8%)	147 (53.8%)	0.066	301 (52.3%)
* BRCA2*	136 (44.9%)	124 (45.4%)		260 (45.1%)
* BRCA1* and *BRCA2*	7 (2.3%)	1 (0.4%)		8 (1.4%)
Do not know or not sure	6 (2.0%)	1 (0.4%)		7 (1.2%)
Time in years since gene mutation identified; mean (SD), median (range)	6.11 (4.7) 6 (0**‒**23)	3.49 (3.5) 2 (0**‒**16)	<0.001	4.87 (4.35) 4 (0‒23)
IES total score by category				
Sub-clinical (0‒8)	166 (54.8%)	144 (41.8%)	0.002	280 (48.6%)
Mild (9‒25)	102 (33.7%)	100 (36.6%)		202 (35.1%)
Moderate (26‒43)	27 (8.9%)	47 (17.2%)		74 (12.8%)
Severe (≥ 44)	8 (2.6%)	12 (4.4%)		20 (3.5%)
Family history^b^				
First-degree relative with breast cancer before age 50 years	221 (72.9%)	197 (72.2%)	0.835	418 (72.6%)
First-degree relative with ovarian cancer at any age	144 (47.5%)	126 (46.2%)	0.742	270 (46.9%)
≥2 family members with breast cancer on the same side of the family	202 (66.7%)	181 (66.3%)	0.926	383 (66.5%)
Male relative with breast cancer	26 (8.6%)	15 (5.5%)	0.150	41 (7.1%)
Breast, ovarian, and/or pancreatic cancer on the same side of the family	112 (37.0%)	111 (40.7%)	0.363	223 (38.7%)
≥3 relatives with breast cancer at any age	140 (46.2%)	126 (46.2%)	0.990	266 (46.2%)
None of the above	14 (4.6%)	9 (3.3%)	0.418	23 (4.0%)
Risk-reducing strategies (completed/current)				
RRBM (only)	46 (15.2%)	44 (16.1%)	0.757	90 (15.6%)
BSO (only)	60 (19.8%)	47 (17.2%)	0.426	107 (18.6%)
RRBM and BSO	95 (31.4%)	98 (35.9%)	0.249	193 (33.5%)
Neither RRM or BSO	102 (33.7%)	84 (30.8%)	0.458	186 (32.3%)

*RRBM*  risk-reducing bilateral mastectomy, *BSO* bilateral salpingo oophorectomy.

^a^Percentage of respondents who answered the question, does not account for missing observations.

^b^First-degree relative: mother, daughter, sister, father, son or brother.

The mean cancer-related distress score, as measured by the IES, was 12.7 (SD = 13.1). Ninety-four participants (16.3%) scored within the moderate to severe range of total cancer-related distress and 280 participants (48.6%) scored in the sub-clinical range (no distress). The prevalence of moderate or severe cancer-related distress is presented in Table 2 for various subgroups.

**Table 2:** Prevalence of moderate or severe cancer-related distress.

Category	Prevalence, *n* (%)
Total	94 (16.3%)
Age (years)	
25–39 (*n* = 254)	46 (18.1%)
40–55 (*n* = 322)	48 (14.9%)
Country	
US, FORCE (*n* = 273)	59 (21.6%)
US, Creighton University (*n* = 38)	2 (5.3%)
UK (*n* = 117)	18 (15.4%)
Canada (*n* = 31)	2 (6.5%)
Australia (*n* = 117)	13 (11.1%)
Recruitment source	
Online through FORCE (*n* = 273)	59 (21.6%)
Clinic (*n* = 303)	35 (11.6%)
Risk-reducing surgery	
None (*n* = 186)	41 (22.0%)
RRBM only (*n* = 90)	14 (15.6%)
BSO only (*n* = 107)	17 (15.9%)
RRBM and BSO (*n* = 193)	22 (11.4%)
Time since genetic testing (years)	
0–1 (*n* = 148)	39 (26.4%)
2–4 (*n* = 176)	31 (17.6%)
5+ (*n* = 252)	24 (9.5%)

Sixteen per cent of the participants had a previous RRBM only, 18.6% had undergone BSO only and 33.5% had both RRBM and BSO. Thirty-two per cent of the participants had neither preventive surgery. The prevalence of moderate or severe cancer-related distress was 22.0% in women without preventive surgery compared to 11.4% in those who had undertaken both surgeries (*P* value = 0.007).

Distress levels were similar across countries. In the USA, 61 of 311 women (19.6%) experienced moderate to severe distress. Distress was more common among American women recruited through the online advocacy group FORCE compared to women recruited through a cancer genetics clinic in Omaha (21.6% versus 5.3%, *P* value < 0.001).

In the multivariable analysis higher education was protective of moderate or severe distress (odds ratio (OR) = 0.57, 95% confidence interval (CI): 0.34–0.96, *P* value = 0.036) (Table 3). Women who had a sister or mother with cancer were not more likely to have moderate or severe distress compared to women with no affected first-degree relative. Women with both RRBM and BSO were less likely to have moderate or severe cancer-related distress compared to women without either surgery (OR = 0.37, 95% CI: 0.18–0.76, *P* value = 0.007); however, those with a single surgery were not different than those with neither surgery. Women recruited through FORCE were more than twice as likely to have moderate or severe levels of cancer-related distress compared to women recruited through cancer genetics clinics in the entire study group (OR = 2.26, 95% CI: 1.34–3.82, *P* value = 0.002). Outside the FORCE group, only one clinic in the Midwest USA provided study subject and at this clinic distress was very low (5.3%), but the sample size was small (*n* = 38).

**Table 3:** Multivariate logistic model evaluating predictors of cancer-related moderate-to-severe cancer-related distress, full sample.

Variables	All women (*n* = 576)		
	Odds ratio	95% CI	*P* value
Intercept	0.30*	(0.14, 0.67)	0.0030
Age			
<40 years	1.00		
40 years or over	1.03	(0.56, 1.91)	0.9222
First-degree relative with breast cancer			
No	1.00		
Yes	1.09	(0.66, 1.79)	0.7306
First-degree relative with ovarian cancer			
No	1.00		
Yes*	1.07	(0.60, 1.90)	0.8298
Higher education			
No	1.00		
Yes	0.57*	(0.34, 0.96)	0.0356
Married			
No	1.00		
Yes	0.85	(0.47, 1.54)	0.5904
Time since genetic test (years)	0.92*	(0.85, 0.98)	0.0158
Have children			
No	1.00		
Yes	1.69	(0.93, 3.08)	0.0863
Cancer risk-reducing surgery			
None	1.00		
RRBM only	0.60	(0.30, 1.21)	0.1537
BSO only	0.54	(0.25, 1.14)	0.1055
RRBM and BSO	0.37*	(0.18, 0.76)	0.0069
Recruitment source			
Clinic	1.00		
Online through FORCE	2.26*	(1.34, 3.82)	0.0023

*P value < 0.05.

**Fig. 1:**
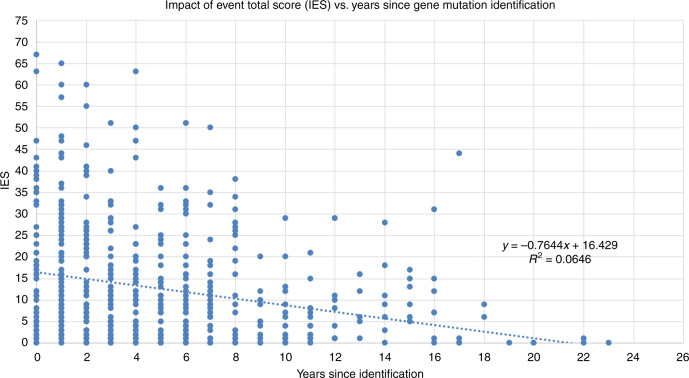
Scatter plot and regression line for Impact of Event Scale (IES) total score and years since gene mutation identification. y-axis: Impact of Event Scale total score. x-axis: Years since identification of gene mutation.

More years since genetic testing were associated with lower levels of cancer-related distress (Fig. 1). For each year elapsed since testing, the probability of experiencing moderate or severe distress declined by 8% (OR = 0.92, 95% CI: 0.854–0.984, *P* value = 0.016).

## Discussion

In this large international study, we report that after an average of 5 years after genetic testing, most women with a *BRCA* mutation without cancer did not have elevated levels of distress. However, a significant proportion (16.3%) did have moderate or severe levels of cancer-related distress. Women with less education were more likely to experience distress than those with post-secondary education.

Since the introduction of genetic testing for *BRCA1* and *BRCA2* over 20 years ago, the psychosocial consequences of genetic testing have received considerable attention.^1–6^ The majority of research has focussed on short-term levels of distress and has suggested that distress may increase immediately following the receipt of positive genetic test results, but levels of distress return to baseline levels or below over time.^4,6^ In the current cross-sectional study in which women were surveyed on average of 5 years post-receipt of genetic test results, distress was significantly lower as time since genetic testing increased. This is consistent with a previous American study that measured long-term cancer-related distress in 107 unaffected BRCA mutation carriers.^2^ This suggests that support should be focussed in the time period immediately following receipt of test results. In an American study, psychosocial telephone counselling shortly after standard genetic counselling was shown to offer modest short-term benefits for distress and anxiety in women with a *BRCA1* or *BRCA2* mutation.^12^ Further research to evaluate psychosocial interventions is required to support women who continue to experience distress in both the short and long term.

Most previous studies have reported on predictors of cancer-related distress in women from single institutions and have included women with cancer.^1–5^ Both cancer and genetic status have been shown to raise cancer-related distress.^13^ We chose to focus on unaffected women with a positive *BRCA1* or *BRCA2* genetic test result to determine if there were modifiable predictors that could be the targets of interventions to reduce cancer-related distress in this subgroup. Although not modifiable, education level and time since testing may help identify women who may require additional support after receiving a positive *BRCA* genetic test result.

The National Comprehensive Cancer Network recommends that *BRCA* mutation carriers have BSO between the ages of 35 and 40 years or when child-bearing is complete, and that RRBM is discussed as an option. In the current study, women who elected for both BSO and RRBM were significantly less likely (OR = 0.37, 95% CI: 0.18–0.76, *P* value = 0.007) to have moderate or severe cancer-related distress compared to women with neither surgery; however, no significant reduction in distress was seen with only one surgery (either BSO or RRBM). We have previously reported on changes in cancer-related distress in unselected Jewish women who were found to have a *BRCA1* or *BRCA2* mutation.^14^ Cancer-related distress decreased significantly after uptake of both bilateral prophylactic mastectomy and BSO in women with a *BRCA1* or *BRCA2* mutation. The current study provides further evidence that risk-reducing surgery is beneficial in reducing cancer-related distress in women with a *BRCA1* or *BRCA2* mutation.

Much of the previous research reporting on psychosocial outcomes after genetic testing for *BRCA1* and *BRCA2* has enrolled women from academic cancer genetics clinics. It is important to evaluate outcomes in women who are recruited from outside cancer genetics clinics as this is increasingly common. In the current study, 21.6% of the women who were recruited through an advocacy group (FORCE) were experiencing moderate or severe levels of cancer-related distress; they were more than twice as likely to experience moderate or severe cancer-related distress than women recruited through cancer genetics clinics (OR = 2.26, 95% CI: 1.34–3.82, *P* value = 0.002). There were only 38 women enrolled from the USA from clinics other than FORCE and the comparison group of American women is small. The reason(s) for this difference are unclear, nor is the direction of the association. These women might not have received traditional genetic counselling and may remain with unresolved uncertainties that provoke anxiety. Alternatively, women who are experiencing cancer-specific distress may seek information about their condition. It is also possible that participating in the online group has the effect of reinforcing the woman’s awareness of her cancer vulnerability on a daily basis and thus changes the affect of the patient. We do not have the data to conclude that the increased levels of distress were the direct consequence of participating in the online forum and this is a topic of future study.

There are several limitations to the current study. This study was a convenience sample and employed a cross-sectional design, and as a result, we were not able to measure changes in distress over time. We only measured distress using one instrument (IES); however, this measure has demonstrated concurrent and discriminative validity in women at an increased risk of developing breast cancer.^15^ In addition, as expected with a survey study, the participation rate was not optimal and we only surveyed women between the ages of 25 and 55 years, so the results may not be generalisable to women over the age of 55 years. Furthermore, we did not collect data about access to care, including insurance status, rural versus urban or access to a genetic counsellor. All the factors should be considered in future research to determine if these factors have an impact on cancer-related distress after receipt of positive BRCA genetic test results.

Those of us who have counselled unaffected women with mutations have noted the wide range of emotions expressed by these women and will have encountered a small number of women who become fixated on their genetic status. Often, these women seek the input of multiple counsellors and physicians. It is not clear if any current cognitive therapies are effective in reducing life-altering levels of distress, but there is some evidence that preventive surgery is therapeutic in this regard. These women may require more targeted support than is available through an online support group or through classical genetic counselling. Further research is required to determine if any additional interventions are effective in this vulnerable subgroup of patients.

## Data Availability

The data that support the findings of this study are available from the corresponding author upon reasonable request.
